# Mottled Raynaud’s phenomenon and hand-arm vibration syndrome: followed up for 10 years

**DOI:** 10.1136/bcr-2023-257314

**Published:** 2024-01-17

**Authors:** Harukazu Hirano

**Affiliations:** Koyo Seikyo Clinic, Fukui Health Cooperative Association, Fukui, Japan

**Keywords:** Occupational and environmental medicine, Dermatological, General practice / family medicine

## Abstract

Vibration white finger is a form of secondary Raynaud’s phenomenon (RP) caused by the use of handheld vibrating tools. RP usually appears on the extremities of the fingers, and its borders are well recognised. No reports have been published on ‘mottled’ RP in continuous observation from the onset to the disappearance of RP. A man in his 60s who had been using vibrating tools such as jackhammers and tampers for 30 years presented with sensations of coldness, burning and numbness. Whole-body cold exposure was performed outdoors in winter, and RP was photographed continuously. ‘Mottled’ RP can be defined as triphasic colour changes: white, blue and red. The patient was taken off work, kept warm and medicated. His symptoms improved slightly after 10 years of follow-up, but the RP did not disappear. ‘Mottled’ RP is rare and refractory and should be recognised as a form of RP.

## Background

Hand-arm vibration syndrome (HAVS) is a common but underdiagnosed occupational disease caused by the use of handheld vibrating tools.^1^ HAVS has three components, namely, peripheral circulation, nerves and the musculoskeletal system. The circulatory disorder, also known as vibration white finger (VWF), is a form of secondary Raynaud’s phenomenon (RP) and the most well-established manifestation of HAVS.^2^ The more advanced stages of HAVS result in disability at work and reduced quality of life.^3^ The prevalence of RP ranges from 2% in temperate regions to 16% in cold climates. RP is a common vasospasm condition (primary RP).^4^

RP may develop secondary to a variety of underlying conditions, including drugs, connective tissue disorders,^5^ and environmental agents and injuries such as HAVS (secondary RP). Secondary RP accounts for 10%–20% of all cases of RP.^6^ RP is triggered by cold exposure, emotional stressors, tobacco smoke and so on. Cold stimulation activates α2-adrenergic receptors in vascular smooth muscle, resulting in cutaneous blood vessel spasms.^7^

RP is classically manifested as triphasic colour changes: white/pallor due to vasoconstriction, blue/cyanosis due to sequestration of deoxygenated blood and red due to reperfusion and hyperaemia.^6^ However, all the three-colour changes are rarely visible to the patient or clinician. A rare phenomenon in which three-colour changes can be recognised continuously in a short time is defined as ‘mottled’ RP. ‘Mottled’ RP has been known empirically,^8^ but no photographic reports have been published on continuous observation from the onset to the disappearance of RP.

We present a rare case of ‘mottled’ RP and HAVS. The cold-water immersion test was performed on the hands to confirm the diagnosis of VWF. However, RP rarely appeared during the examination. Additionally, whole-body cold exposure, which is more sensitive than partial exposure to cold, was performed.^9^

## Case presentation

**Figure 1:**
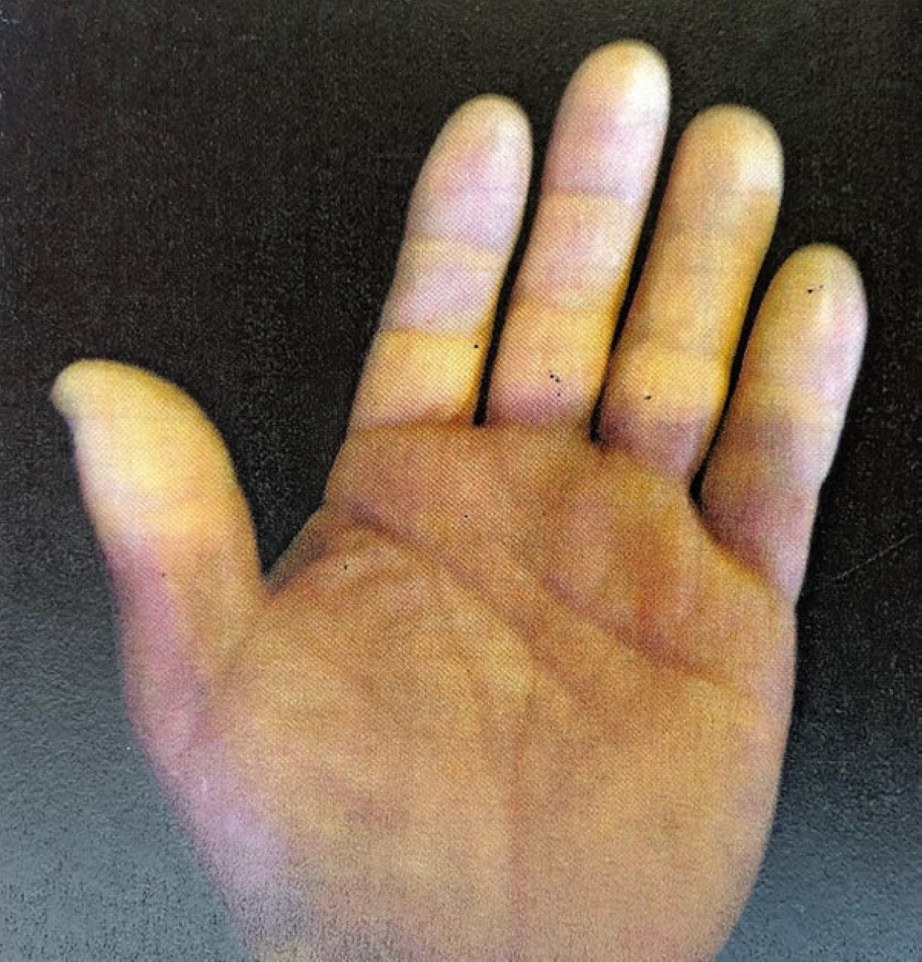
A photograph of Raynaud’s phenomenon (RP) taken by the patient with his mobile phone. RP was observed in the five fingers of the left hand, but the photograph was blurred and unreliable.

A man in his 60s presented to the local occupational medicine specialist complaining of progressively increasing sensations of coldness, burning and numbness in his hands, but not in the lower extremities. He had no smoking or drinking habits and no underlying diseases. The patient worked in civil engineering for 30 years using handheld vibrating tools such as jackhammers, tampers and chipping hammers. He had no family history of neurological disease, cardiovascular disease or RP. He showed a photograph of RP (figure 1), taken by himself with his mobile phone, but it was blurred and unreliable.

Visual inspection of the skin of the extremities showed no abnormal findings. Physical examination revealed palpable dorsalis pedis and radial artery pulsations. Additionally, no vascular murmurs were audible in the neck or abdomen. Upper and lower extremity tendon reflexes were normal. Hoffmann, Trömner and Wartenberg’s signs were negative. Tinel’s sign was positive for carpal tunnel syndrome and negative for cubital tunnel syndrome. Sensory tests, including a pain test using the tip of a safety pin and a tactile test using cotton, unveiled significant impairment in both hands, with a pronounced impact on the fingertips. The fingertip vibration threshold test was conducted at 125 Hz using a measuring device that adheres to ISO standards. Although both fingers exhibited severe impairment, the skin temperature at the time of measurement was approximately 21°C, which could have influenced the neurosensory test results. No muscle atrophy was observed in the trunk or extremities.

## Investigations

Laboratory findings (table 1) were negative for antinuclear, anti-Scl-70 and anticentromere antibodies. The ECG and ankle-brachial pressure index were normal. Nerve conduction velocity studies were performed, and mild conduction gaps were observed in the carpal tunnel, but not in the cubital tunnel. His X-ray showed moderate osteoarthritis in the finger joint, but not in the hand and elbow joints. A cervical spine X-ray showed a narrowing of the intervertebral space. A cervical spine MRI showed no compression on nerve roots or abnormal signals in the cervical spinal cord.

**Table 1:** Laboratory findings

Laboratory value	Results	Reference
Haemoglobin, g/dL	15.2	13.6–18.3
White cell count, /μL	4000	3500–9700
Platelets, 10^4^ /µL	14.5	14.0–37.9
Glucose, mg/dL	104	70–109
Aspartate aminotransferase, U/L	20	13–30
Alanine aminotransferase, U/L	11	10–42
Alkaline phosphatase, U/L	79	38–113
Creatinine, mg/dL	0.75	0.65–1.07
Blood urea nitrogen, mg/dL	18	8–20
C reactive protein, mg/dL	0.01	0.0–0.14
Thyroid-stimulating hormone, μIU/mL	1.81	0.5–5.0
Free T4 hormone, ng/dL	1.42	0.9–1.7
Rheumatoid factor	Negative	0–15
Antinuclear antibodies	Negative	0–1
Anti-Scl-70 antibodies, U/mL	Negative	0–6.9
Anticentromere antibodies, U/mL	Negative	0–6.9

In this case, the cold-water immersion test for HRVS was conducted by immersing the subject in cold water at 10°C for 10 min, a method commonly used in Japan.^10^ The ambient room temperature was maintained at 21°C±1°C. We measured the skin temperature of the finger pad (distal phalanx area) before and at 5 and 10 min after immersion in cold water. This test deviates from the International Organization for Standardization (ISO) 14835-1 standard, which recommends immersion in cold water at 12°C for 5 min.

Whole-body cold exposure was performed outdoors in winter to confirm RP. This test aims to demonstrate distinct RP. The presence or absence of RP is important for diagnosing work-related injuries. The test was performed while the patient was not wearing a warm coat or gloves. The test was performed two times: on January 11 (−2°C outside temperature) from 6:04 to 6:36 hours and on January 16 (−3°C outside temperature) from 6:19 to 6:49 hours. RP appeared after about 20 min, and a picture of RP was taken by the author.

**Figure 2:**
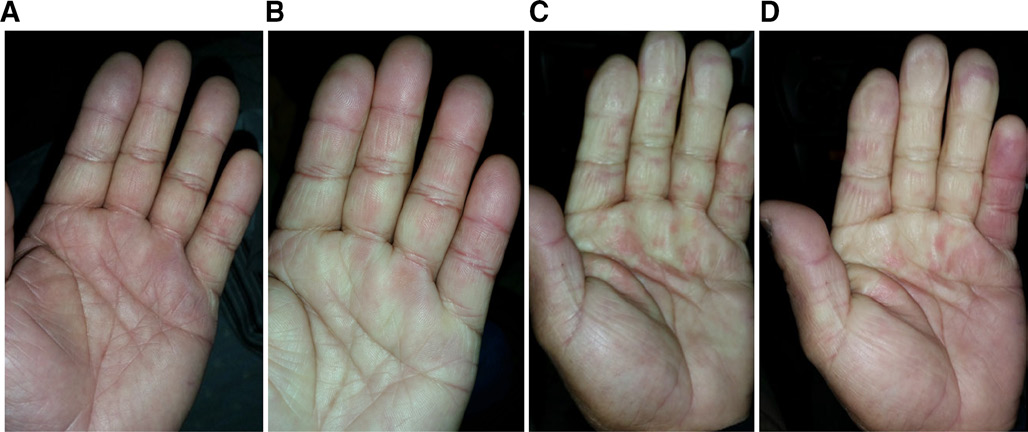
RP after the first whole-body cold exposure. Before RP, the fingers were slightly discoloured (A), but after RP development, the whiteness of the palm was conspicuous (B). RP magnified with fingers, and patchy red spots appeared on the palms, becoming mottled (C). During the recovery phase of RP, the white part of the fingers decreased, and redness increased in the little finger due to reactive hyperaemia (D). Dark purple (cyanosis) was not seen. RP, Raynaud’s phenomenon.

After the first whole-body cold exposure, RP appeared (figure 2). Before RP development, the fingers were slightly discoloured. Subsequently, the whiteness of the palm became noticeable. With the expansion of RP into the fingers, patchy red spots appeared on the palms and became ‘mottled’. In the recovery phase, increased redness was observed in the little finger due to reactive hyperaemia. Dark purple (cyanosis) was not seen.

**Figure 3:**
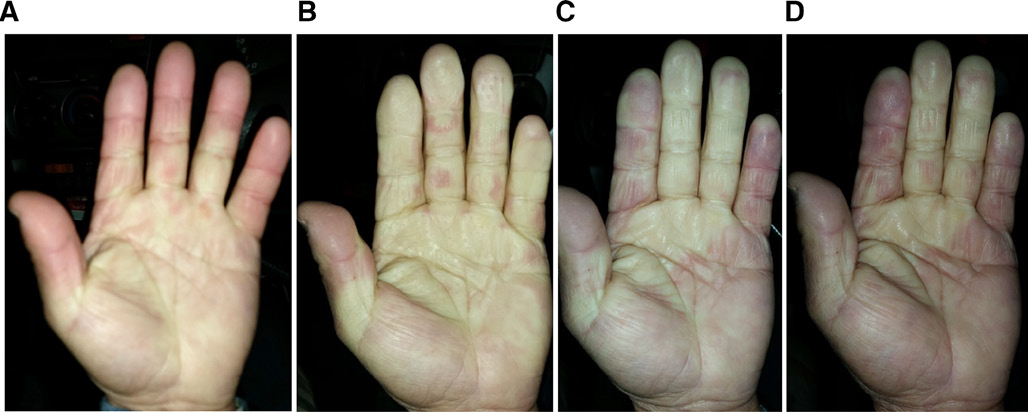
RP after the second whole-body cold exposure. RP appeared from the palm as in the first time (A) and expanded to the fingers other than the thumb (B). During the recovery phase of RP, dark purple (cyanosis) was slightly observed on the little finger (C). RP recovered, and the index finger was cyanotic (D). During the RP regression phase, the palms and fingers became ‘mottled’. RP, Raynaud’s phenomenon.

RP also appeared after the second whole-body cold exposure (figure 3). As in the first time, RP appeared from the palm and expanded to fingers other than the thumb. During the recovery phase of RP, dark purple (cyanosis) was observed on the index finger, followed by the little finger. During the RP regression stage, the palms and fingers became ‘mottled’.

## Differential diagnosis

Clinical examination ruled out other conditions presenting with RP, including connective tissue diseases such as scleroderma and lupus or mixed connective tissue diseases. The patient had no history of use of any drugs that cause secondary RP, including nicotine, ergotamine, beta-blockers and cocaine. Additionally, no hypothyroidism or malignant disease was found. Buerger’s disease and arteriosclerosis obliterans were excluded.

Neurological examination and cervical spine MRI showed no evidence of radiculopathy or cervical myelopathy. This case might be complicated by carpal tunnel syndrome. Carpal tunnel syndrome is a common complication of HAVS^11^ and is also well known to present with RP.^12^ However, it was difficult to diagnose these symptoms with carpal tunnel syndrome alone because the numbness was stronger in the 10 fingertips and extended to both hands. The most common neurological symptoms of HAVS, as in this case, are tingling, numbness and pain in the symmetrical distribution of the glove, caused by small nerve fibre injuries.^13^

Other diseases causing peripheral neuropathy were also excluded, including diabetes, chronic hepatitis, rheumatoid arthritis, hereditary neuropathy, neurofibromatosis and sarcoidosis. In this case, the neurological symptoms were speculated to be a combination of carpal tunnel syndrome and peripheral neuropathy caused by hand-arm vibration. In the cold season, ischaemic symptoms of peripheral circulatory disturbance appeared, and the patient’s pain worsened even more.

## Treatment

The patient was diagnosed with HAVS. A medical certificate was submitted indicating an occupational injury. The local labour office has granted and certified the work injury claim. Employment was discontinued. Treatment was initiated. Mecobalamin (500 mg) three tablets and beraprost sodium (40 µg) three tablets were prescribed for drug treatment.^14^ Alprostadil (10 µg) was given intravenously in winter when RP appeared. The patient received hyperthermia and exercise therapy. Additionally, the patient was advised to make lifestyle improvements such as keeping warm in the cold season, performing moderate exercise and alleviating psychological stress.

## Outcome and follow-up

The patient continued his treatment and was followed up by regular clinic visits. Treatment for cold hands, RP in winter, numbness and dexterity disorder was effective but insufficient, as the symptoms persisted for up to 10 years after diagnosis. The cold-water immersion test was performed each summer and winter to confirm symptoms and assess disease status. This test is required by Japanese law concerning occupational injury. Finger skin temperature was measured using a thermometer (D642, Tateyama Kagaku IND., Japan). The cold-water immersion test also showed no clear improvement in skin temperature until 10 years later (table 2). Emotional distress increased as he had difficulty even doing light work. He continued to receive workers’ compensation.

**Table 2:** The skin temperature of the left ring finger pad was measured before and 5 and 10 min after immersion in cold water

	Before	5 min	10 min
	June/December	June/December	June/December
2012	–/21.1	–/14	–/14.8
2013			
2014	18.6/17.9	16.7/12.1	26.3/13.3
2015	19.2/21.4	12.9/12.5	14.5/13.4
2016	21.8/20.8	13.1/13.0	14.7/14.3
2017	18.6/18.7	13.1/12.9	14.6/13.7
2018	–/18.1	–/12.1	–/13.6
2019	21.6/19.3		
2020	–/20.9		
2021	20.5/17.3		
2022	21.7/19.5		

It was not tested in 2013, and no cold-water immersion test was performed in 2019–2022 due to the spread of COVID-19.

## Discussion

The pathogenesis of HAVS is complicated and not fully understood. It is believed that mechanical trauma and shear stress from hand-arm vibration cause local endothelial damage and dysregulation.^15^ Hand-arm vibration can cause peripheral vasoconstriction by activating the sympathetic nervous system. Furthermore, erythrocyte and leucocyte activation probably contribute to vasospastic paroxysms.^16^

Occupational history taking and physical and clinical examinations are performed to rule out alternative diagnoses. Some cases need to be referred to an occupational medicine specialist for further investigation. Management includes reducing or eliminating vibration exposure, avoiding cold conditions, smoking cessation, medication and physical therapy.^17^

Whole-body cold exposure is more sensitive to RP than partial exposure, such as hand immersion in cold water.^9^ Whole-body cold exposure testing is painful for participants and is rarely performed in clinical settings. In this case, cold-water immersion tests were performed annually up to 10 years after diagnosis. As a detailed examination, cold stress finger systolic blood pressure test,^18^ angiography^15^ and label-free photoacoustic imaging^19^ can be performed by an occupational medicine specialist to evaluate VWF. However, they are not used in clinical settings due to the high cost or invasiveness of the devices. The presence or absence of VWF depends on whether whole body or partial exposure occurred, and VWF is strongly influenced by environmental temperature. The VWF has not been reported in tropical countries. Finger coldness may be an important surrogate for vascular disorders in tropical environments.^20^

The Stockholm Workshop Scale is used worldwide to assess cold-induced RP in HAVS.^21^ This staging system—stage 0 and 4 stages^1–4^ with cold-induced attacks of RP—has increased clinical utility. This case was stage 3, which is the most severe stage. In the subsequent new international consensus criteria, stage 4 in SWS was omitted from ICC staging. This decision was made because stage 4 was not attributed to the vibration effects and likely indicated an underlying medical condition.^14^

Patients may be aware of RP. However, it is rarely seen by clinicians because the symptoms are transient. With the recent spread of mobile phones, it has become possible to easily take pictures and confirm RP.

It is necessary to keep watching RP for a certain period to confirm the three phases. RP is rarely reported in papers. To our knowledge, this is the first report of mixed triphasic ‘mottled’ RP caused by whole-body exposure to cold. ‘Mottled’ RP is thought to be caused by temporally and spatially variable vasospasms in the fingers and hands. Laser Doppler flowmetry can be used to demonstrate abnormal cutaneous microvascular reactivity to central and local axonal reflex sympathetic stimulation.^22^ ‘Mottled’ RP is expected to appear in cases where the cutaneous microvascular circulation exhibits variable hyper-reactivity to sympathetic nerves. Thus, further studies are needed to elucidate the physiological and biological mechanisms of ‘mottled’ RP. As in this case, patients with atypical skin symptoms are often immediately referred to a rheumatologist without proper consideration of non-rheumatic diseases, including diseases caused by exposure to physical agents. In the clinical setting, determining the social work history is crucial.^23^.

In this case, routine skin temperature testing between summer and winter showed no improvement. Therapeutic efficacy in severe cases is limited, so early diagnosis and occupational health interventions are critical. Patients often do not mention RP unless the clinician asks about it. Additionally, ‘mottled’ RP is rarely identified by clinicians and is often dismissed as atypical RP. Patients with ‘mottled’ RP may have severe circulatory impairment, and it should be recognised as a form of RP.

**Patient’s perspective**

I have been working in civil engineering since my early twenties and have used vibration tools such as jackhammers, breakers, and tamper for 30 years. In the late thirties, my hands were cold, and tingling, so I could not sleep. I visited a hospital, but the doctors did not make a definite diagnosis. In the fifties, I was surprised to find that my fingers were white when I took off my gloves. Despite my symptoms, I continued to work to ensure my livelihood.

With the increasing age, symptoms gradually worsened, and the frequency of white fingers increased. It is hard to describe the numbness in words, but it is always a tingling numbness, like a bunch of ants crawling around. I have a poor sense of holding tools at work, and sometimes I drop a shovel or a hammer. I also experienced difficulties in daily life, such as dropping chopsticks, using toilet paper, and being unable to write properly.

I underwent various medical tests and was diagnosed with hand-arm vibration syndrome. I know an energetic colleague who is still working at 70. I was at the limit of my ability to work, so I decided to receive workers’ compensation while receiving treatment. The treatment effect is temporary, and my symptoms continue. The frequency of white fingers has decreased, but it also appears in winter.

Life is hard, and I try to buy cheap food. I never drank alcohol, stopped smoking and never gambled. I often have trouble sleeping, do not get along with my wife, and cannot feel hope for the future.

**Learning points**

- Raynaud**’**s phenomenon (RP) is found in 2%–16% of the general population. Secondary RP caused by connective tissue diseases is well known.
- RP is recognised by handheld vibration tool users and named vibration white finger. Occupational history taking is important.
- ‘Mottled’ RP is defined as triphasic colour changes (white, blue and red) of RP. It appears to be seen in severe cases and should be recognised as a form of RP.
